# Zbtb38 transcriptionally activates *XIAP* to regulate apoptosis in development and cancer

**DOI:** 10.1093/jmcb/mjag011

**Published:** 2026-03-17

**Authors:** Toshiaki Shigeoka, Hiroyuki Nagaoka, Nunuk Aries Nurulita, Shogo Tada, Yasumasa Bessho, Yasumasa Ishida, Eishou Matsuda

**Affiliations:** Functional Genomics and Medicine, Nara Institute of Science and Technology, Ikoma 630-0192, Japan; Functional Genomics and Medicine, Nara Institute of Science and Technology, Ikoma 630-0192, Japan; Faculty of Pharmacy, University of Muhammadiyah Purwokerto, Jalan Raya Dukuhwaluh PO BOX 202, Purwokerto 53052, Indonesia; Functional Genomics and Medicine, Nara Institute of Science and Technology, Ikoma 630-0192, Japan; Gene Regulation Research, Nara Institute of Science and Technology, Ikoma 630-0192, Japan; Functional Genomics and Medicine, Nara Institute of Science and Technology, Ikoma 630-0192, Japan; Functional Genomics and Medicine, Nara Institute of Science and Technology, Ikoma 630-0192, Japan

**Keywords:** apoptosis, XIAP, Bcl-2, embryonic stem cell differentiation, embryonic development, cancer

## Abstract

The X-linked inhibitor of apoptosis protein (XIAP) is a key suppressor of apoptosis, crucial for cellular differentiation, embryogenesis, and cancer progression. However, its upstream regulatory mechanisms remain poorly understood. Here, we demonstrate that the zinc finger transcription factor Zbtb38, a negative regulator of apoptosis, modulates *XIAP* expression in both loss- and gain-of-function experiments, irrespective of p53 expression. Notably, XIAP overexpression rescues the apoptosis induced by *Zbtb38* knockdown, indicating that Zbtb38-associated apoptosis is at least partially XIAP-dependent. Mechanistically, Zbtb38 binds to E-box motifs within upstream regulatory regions of *XIAP* and activates its transcription. During embryonic stem cell differentiation and embryogenesis, *Zbtb38* depletion increases apoptosis and reduces *XIAP* and *Bcl-2* expression, underscoring their functional relevance in these processes. Analysis of human tumor datasets reveals a strong positive correlation between *ZBTB38* and *XIAP* expression, with elevated *ZBTB38* levels associated with high-grade malignancies. Furthermore, *Zbtb38* knockdown induces apoptosis in cancer cells with reduced *XIAP* expression, regardless of p53 expression. Collectively, these findings uncover a novel Zbtb38–XIAP axis that regulates apoptosis during cellular differentiation, development, and oncogenesis and highlight its therapeutic potential in *XIAP*-driven and *p53*-deficient tumors.

## Introduction

In mammals, apoptosis is a genetically programmed form of cell death essential for maintaining tissue homeostasis by eliminating damaged or unnecessary cells (Elmore, 2007; Nagata, 2018). It also contributes to immune surveillance by removing abnormal or neoplastic cells. Intrinsic apoptosis, also known as mitochondrial apoptosis, is triggered by internal signals such as DNA damage, hypoxia, hydrogen peroxide, and survival factor deprivation (Bock and Tait, 2020; Lossi, 2022). These stimuli activate the initiator caspase-9, which is cleaved into its active form (cCasp9) and subsequently activates downstream effector caspases, including caspase-3 and caspase-7 (cCasp3 and cCasp7) (Larsen and Sørensen, 2017; Sahoo et al., 2023). This caspase cascade results in the cleavage of various cellular substrates, with cleaved poly(ADP-ribose) polymerase (cPARP) serving as a hallmark of irreversible apoptosis (Soldani et al., 2001).

The Bcl-2 family of proteins plays a central role in regulating the release of apoptogenic proteins from the mitochondria. This family includes anti-apoptotic proteins (e.g. Bcl-2, Bcl-xL, and Bcl-w), BH3-only pro-apoptotic proteins (e.g. Bik, Bad, Noxa, and Puma), and multidomain pro-apoptotic proteins (e.g. Bax and Bak) (Czabotar and Garcia-Saez, 2023). BH3-only proteins promote apoptosis by binding to and inhibiting anti-apoptotic proteins (Siddiqui et al., 2015; Kale et al., 2018). Bcl-2, a well-characterized anti-apoptotic protein, sequesters Bax and Bak to prevent cytochrome c release from mitochondria (Larsen and Sørensen, 2017; Lossi, 2022). Another key regulator is p53, which promotes apoptosis by transcriptionally activating pro-apoptotic genes (e.g. *Noxa* and *Puma*), repressing anti-apoptotic genes (e.g. *Bcl-2* and *Bcl-xL*), and interacting directly with mitochondrial membranes (Hao et al., 2022). These multifaceted functions underscore p53’s pivotal role as a tumor suppressor in cancer (Aubrey et al., 2018). In contrast to the Bcl-2 family proteins, the inhibitor of apoptosis proteins (IAPs) suppress apoptosis by directly inhibiting caspases. The IAP family comprises three classes: Class I (cIAP1, cIAP2, and X-linked inhibitor of apoptosis protein [XIAP]), Class II (NAIP), and Class III (survivin and Bruce) (Liston et al., 1997; Deveraux and Reed, 1999). Class I IAPs contain baculovirus IAP repeat (BIR) domains that directly bind to and inhibit caspases. Notably, XIAP uniquely inhibits caspases at physiological concentrations, distinguishing it from cIAP1 and cIAP2 (Dohi et al., 2004; Dubrez-Daloz et al., 2008). While single knockout (KO) mice for *XIAP, cIAP1*, or *cIAP2* and *XIAP*/*cIAP2*, double KO mice are viable and fertile, double KO mice for *XIAP*/*cIAP1* or *cIAP1*/*cIAP2* exhibit embryonic lethality around embryonic day 10.5 (E10.5), indicating functional redundancy (Harlin et al., 2001; Moulin et al., 2012; Heard et al., 2015). IAP inhibitors such as Smac and Xaf1 displace caspases by binding to IAP BIR domains (Dynek and Vucic, 2013), while Htra2 promotes IAP degradation through its serine protease activity (Verhagen et al., 2002). Despite XIAP’s central role in apoptosis inhibition, its upstream regulatory mechanisms remain poorly defined.

We and others have shown that Zbtb38, also known as CtBP-interacting BTB zinc finger protein (CIBZ), binds to both methylated and unmethylated DNA through its first zinc finger cluster (ZF1–5), functioning as either a transcriptional activator or a repressor depending on the chromatin context and target gene (Sasai et al., 2005, 2010; Filion et al., 2006; Oikawa et al., 2011; de Dieuleveult and Miotto, 2018). Our previous work demonstrated that Zbtb38 promotes the proliferation of mouse embryonic stem (ES) cells while inhibiting their differentiation toward the mesodermal lineage (Nishii et al., 2012; Kotoku et al., 2016). We also reported that siRNA-mediated knockdown of *Zbtb38* induces apoptosis in murine cells, as evidenced by increased levels of cCasp9, cCasp3, cCasp7, and cPARP, along with enhanced Annexin V uptake (Oikawa et al., 2008). Consistently, Zbtb38 overexpression suppresses apoptosis and restores spinal cord function after injury in mice (Cai et al., 2017). Recently, we showed that heterozygous loss of *Zbtb38* results in increased apoptosis shortly after implantation, leading to early embryonic lethality at E9.5 (Nishio et al., 2022). Despite these findings, the molecular mechanism by which Zbtb38 regulates apoptosis remains unclear.

In this study, we investigated the downstream targets and regulatory mechanisms of Zbtb38 in apoptosis. Comparative expression analyses in *p53*-expressing and *p53*-deficient cells revealed a consistent correlation with *XIAP*. Sequence analysis uncovered two conserved E-box motifs upstream of the *XIAP* transcription start site (TSS), and chromatin immunoprecipitation (ChIP) and luciferase assays were used to confirm Zbtb38 binding and transcriptional activation. Human tumor datasets showed a strong *ZBTB38*–*XIAP* correlation, with elevated ZBTB38 expression in high-grade tumors. Moreover, Zbtb38 depletion increased apoptosis and reduced *XIAP* and *Bcl-2* expression during ES cell differentiation and embryogenesis. Collectively, these findings indicate that Zbtb38 regulates apoptosis by activating *XIAP* transcription via E-box motifs during cellular differentiation, development, and tumorigenesis.

## Results

### XIAP is identified as a potential direct target of Zbtb38 in apoptosis regulation

**Figure 1:**
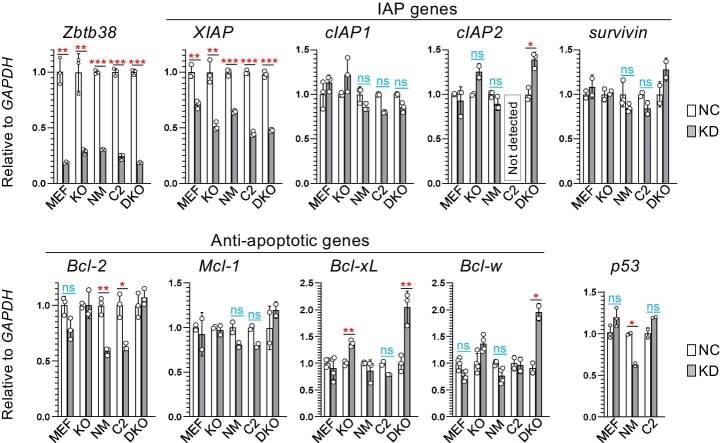
*XIAP* expression correlates with *Zbtb38* expression across all *p53*-expressing and *p53*-deficient cell lines. The mRNA levels of apoptosis-related genes were measured by RT-qPCR in negative control (NC) and *Zbtb38* knockdown (KD) groups. *GAPDH* was used as an internal control, and transcript levels were normalized to *GAPDH* (set as 1). KO, *p53* knockout MEFs; NM, NMuMG; C2, C2C12; DKO, *p53*/*Dnmt1* double knockout MEFs. *P < 0.05, **P < 0.01, ***P < 0.001. ‘ns’ indicates no significance.

Our previous studies demonstrated that siRNA-mediated knockdown of *Zbtb38* induces apoptosis in C2C12 cells and *p53* KO mouse embryonic fibroblasts (MEFs). However, its direct downstream target remains unclear. To address this, we analyzed the effects of *Zbtb38* knockdown in *p53*-expressing (MEFs, NMuMG, C2C12) and *p53*-deficient (*p53* KO and *p53*/*Dnmt1* double knockout [DKO]) cell lines. Since two independent siRNA duplexes targeting distinct regions of *Zbtb38* produced consistent results in preliminary experiments (Oikawa et al., 2008), one was used for subsequent experiments. Reverse transcription-quantitative polymerase chain reaction (RT-qPCR) analysis revealed that *Zbtb38* knockdown consistently reduced *XIAP* mRNA levels across all cell lines, with *Bcl-2* expression being decreased in NMuMG and C2C12 cells (Figure 1). In contrast, the expression levels of other IAP genes (*cIAP1, cIAP2*, and *survivin*), IAP inhibitor genes (*Smac, HtrA2*, and *XAF1*), and additional apoptosis-related genes exhibited minimal or inconsistent changes (Figure 1; Supplementary Figure S1A). For example, *Zbtb38* knockdown upregulated *Bik* in MEFs and *Noxa* in *p53* KO MEFs, while increasing the expression of both anti-apoptotic (*Bcl-xL* and *Bcl-w*) and pro-apoptotic genes (*Bax, Bak, Bik, Noxa*, and *Puma*) in DKO MEFs (Figure 1; Supplementary Figure S1A). These findings suggest that *XIAP* is a likely direct downstream target of Zbtb38.

### Zbtb38 modulates apoptosis through XIAP regulation, independently of p53 expression and DNA methylation

**Figure 2:**
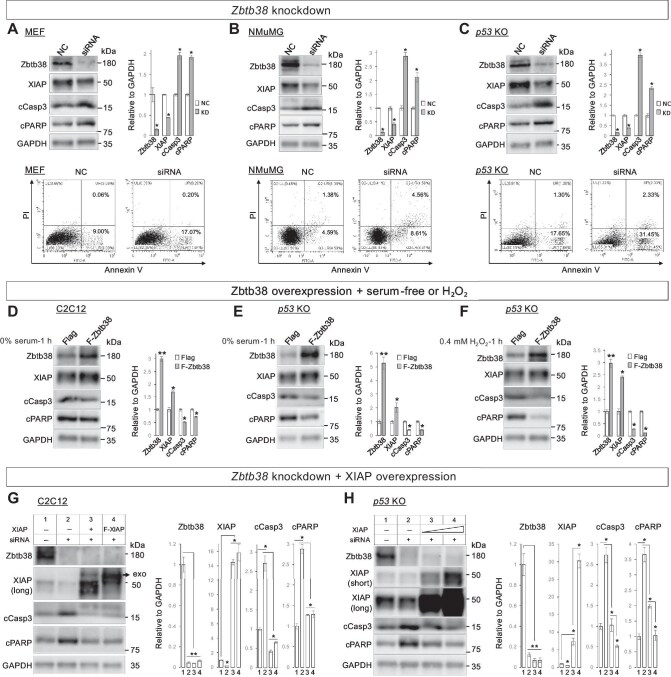
Zbtb38 regulates apoptosis through XIAP independently of p53 expression. (**A**–**C**) *Zbtb38* knockdown induces apoptosis in MEFs (**A**), NMuMG cells (**B**), and p53 KO MEFs (**C**). Cells were transfected with negative control siRNA (NC) or *Zbtb38* siRNA (KD). Upper: protein levels of Zbtb38, XIAP, cCasp3, cPARP, and GAPDH were assessed by immunoblotting. Shown are representative blots from three independent biological replicates and densitometric quantification normalized to GAPDH. Lower: Annexin V/PI staining analyzed by flow cytometry. Representative dot plots from three independent biological replicates are shown. Early apoptotic cells were defined as Annexin V^+^/PI^−^ and late apoptotic/necrotic cells as Annexin V^+^/PI^+^, and their percentages are shown in the lower-right and upper-right quadrants, respectively. (**D**–**F**) Zbtb38 overexpression suppresses apoptosis induced by serum starvation or H₂O₂. C2C12 cells were transfected with Flag or Flag-Zbtb38 (F-Zbtb38) for 36 h, followed by serum starvation for 1 h (**D**). *p53* KO MEFs were transfected with Flag or F-Zbtb38 for 30–36 h, followed by serum starvation for 1 h (**E**) or 0.4 mM H₂O₂ treatment for 1 h (**F**). Protein levels were assessed by immunoblotting and quantified. (**G** and **H**) XIAP overexpression rescues apoptosis induced by *Zbtb38* knockdown. *p53*-proficient (C2C12; **G**) and *p53*-deficient (*p53* KO; **H**) cells were transfected with an empty vector (pcDNA3-2×Flag, lanes 1 and 2) or XIAP expression plasmid (untagged XIAP, lane 3; 2×Flag-tagged XIAP [F-XIAP], lane 4) for 24 h, followed by negative control siRNA (NC, lane 1) or *Zbtb38* siRNA (lanes 2–4) for additional 24 h. Protein levels were assessed by immunoblotting and quantified. Arrows indicate exogenous (exo) XIAP. Data are presented as mean ± SD from two independent biological experiments. *P < 0.05, **P < 0.01.

To further investigate whether *XIAP* is a direct downstream target of Zbtb38, we performed immunoblotting following *Zbtb38* knockdown, overexpression, and rescue experiments. *Zbtb38* knockdown consistently reduced XIAP protein levels and induced apoptosis, as evidenced by increased levels of cCasp3 and cPARP across all examined cell lines, regardless of p53 expression status (Figure 2A–C; Supplementary Figure S1B and C). Notably, *Zbtb38* knockdown induced apoptosis in *p53*/*Dnmt1* DKO MEFs, which lack DNA methylation, demonstrating that its anti-apoptotic function is independent of both p53 and DNA methylation (Supplementary Figure S1C). This pro-apoptotic effect was corroborated by Annexin V/propidium iodide (PI) flow cytometry, which showed increased Annexin V-positive populations (including Annexin V^+^/PI^−^ early apoptotic cells) following *Zbtb38* knockdown (Figure 2A–C). *p53* KO MEFs showed elevated baseline apoptosis (17.65%), consistent with increased genomic instability. *Zbtb38* knockdown further increased this fraction, supporting a p53-independent pro-survival role for Zbtb38. To validate *XIAP* as a functional downstream target, we overexpressed Flag-tagged Zbtb38 (Flag-Zbtb38) in both *p53*-expressing and *p53*-deficient cells and assessed its effect on apoptosis under cellular stress conditions. In C2C12 cells, Flag-Zbtb38 increased XIAP expression and reduced serum deprivation-induced apoptosis compared to controls (Figure 2D). Similarly, in *p53* KO MEFs, Flag-Zbtb38 elevated XIAP expression and inhibited apoptosis triggered by serum deprivation or hydrogen peroxide (H₂O₂) treatment (Figure 2E and F). Consistent with a stress-responsive regulation of this pathway, low-serum exposure in C2C12 cells reduced Zbtb38 concomitantly with XIAP, accompanied by caspase-3 activation, as evidenced by immunoblotting and semi-quantitative PCR (Supplementary Figure S2A and B). Semi-quantitative PCR further confirmed a positive correlation between *Zbtb38* and *XIAP* expression, but not with other apoptosis-related genes, in both Zbtb38-overexpressing and *Zbtb38*-knockdown C2C12 cells (Supplementary Figure S2C and D).

To test whether XIAP mediates Zbtb38’s anti-apoptotic function, we performed rescue experiments. In C2C12 cells, overexpression of either untagged XIAP or Flag-XIAP significantly suppressed apoptosis resulting from *Zbtb38* knockdown (Figure 2G). In *p53* KO MEFs, XIAP overexpression attenuated apoptosis induced by *Zbtb38* knockdown in a dose-dependent manner (Figure 2H). Importantly, *XIAP* overexpression did not alter Zbtb38 expression, confirming *XIAP* as a downstream target. Together, these results demonstrate that Zbtb38 suppresses apoptosis primarily through the upregulation of *XIAP*, independently of p53 expression and DNA methylation.

### Zbtb38 binds to E-box motifs in the XIAP upstream region and activates transcription

**Figure 3:**
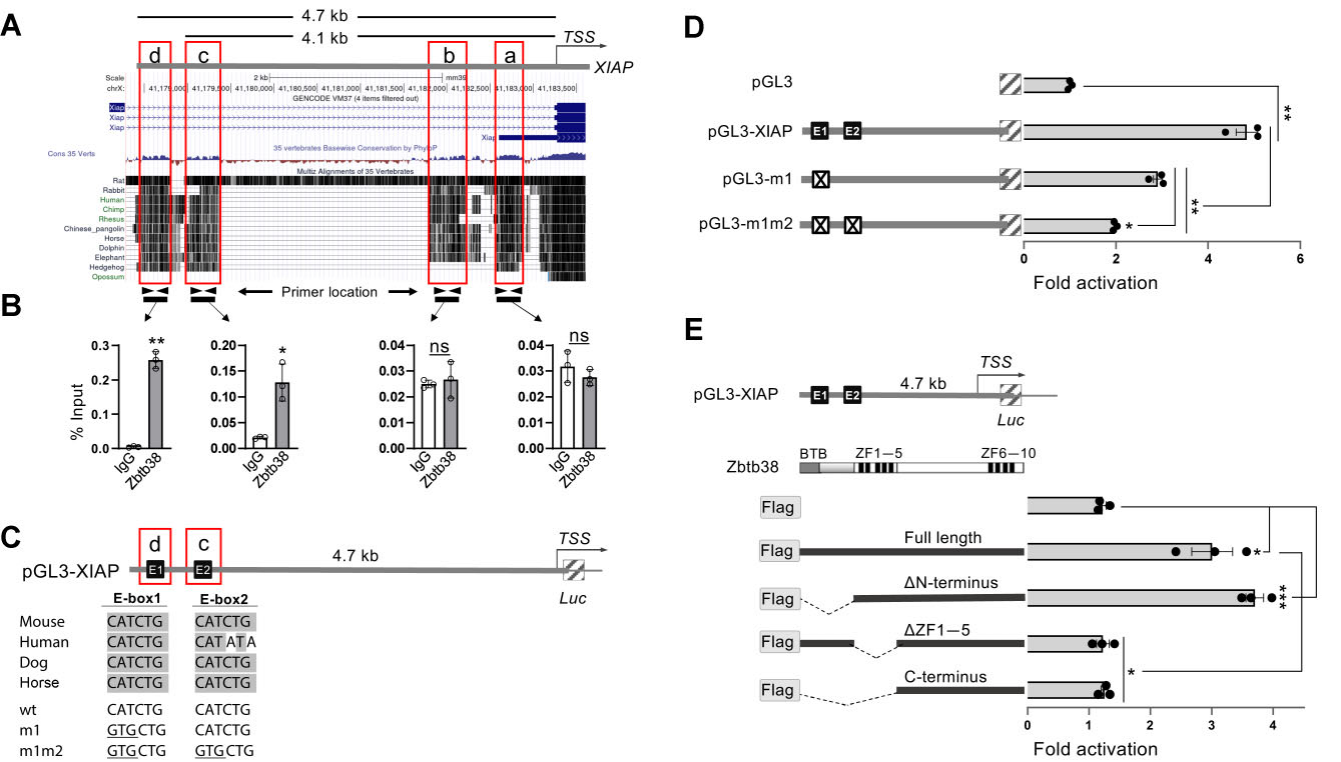
Zbtb38 directly activates *XIAP* transcription via E-box motifs. (**A**) Schematic of the *XIAP* regulatory regions. Squares indicate conserved regions among vertebrate species. Regions a and b represent putative promoter regions; regions c and d represent putative enhancer regions. (**B**) ChIP–qPCR results in C2C12 cells. IgG-precipitated DNA (negative control) and Zbtb38-immunoprecipitated DNA were analyzed by RT-qPCR using primers at the indicated locations. Data are shown as fold enrichment relative to the input control (set to 1). (**C**) Schematic representation of two conserved E-box motifs in the *XIAP* upstream region. Underlined sequences show nucleotide substitutions. wt, wild-type; m1, E-box1 mutant; m1m2, double mutant of E-box1 and E-box2. Luc, luciferase reporter. (**D**) Luciferase assay in HEK293T cells co-transfected with pGL3 or indicated pGL3 constructs and Flag or Flag-Zbtb38. Luciferase activity was normalized to that of pGL3/Flag (set to 1). (**E**) Schematic of Zbtb38 deletion mutants fused to Flag and their transcriptional activation of the pGL3-XIAP 4.7 kb region. Luciferase activity was normalized to that of pGL3/Flag (set to 1). *P < 0.05, **P < 0.01, ***P < 0.001. ‘ns’ indicates no significance.

To investigate whether Zbtb38 binds to the *XIAP* promoter or its upstream regulatory regions, we performed ChIP assays in C2C12 cells. Given the limited understanding of *XIAP* transcriptional regulation in mammals, we focused on four evolutionarily conserved regions upstream of the TSS, designated as regions a–d (Figure 3A). ChIP analysis revealed that Zbtb38 specifically bound to regions c and d, located ∼4.1 kb and ∼4.7 kb upstream of the *XIAP* TSS, respectively, but not to the proximal promoter region a or b (Figure 3B). A similar binding pattern was observed in *p53* KO MEFs (Supplementary Figure S3), indicating that Zbtb38 binding is independent of p53 expression.

To assess the transcriptional activity of Zbtb38 on *XIAP*, we performed luciferase assays in HEK293T and C2C12 cells, which expressed low and high levels of Zbtb38, respectively (Oikawa et al., 2011). Zbtb38 overexpression in HEK293T cells increased the luciferase activity of the *XIAP* 4.7 kb upstream region in a dose-dependent manner, while its knockdown in C2C12 cells suppressed the activity of this region (Supplementary Figure S4). Motif analysis identified two canonical E-box motifs (CANNTG), designated E-box1 (E1) and E-box2 (E2), located within regions c and d, respectively (Figure 3C). Luciferase assays using reporter constructs with point mutations in E1 (m1) or in both E1 and E2 (m1/m2) showed that Zbtb38 strongly activated the wild-type 4.7 kb upstream region, whereas activation was reduced in the m1 mutant and further attenuated in the m1/m2 double mutant (Figure 3D). These results indicate that both E-boxes contribute to Zbtb38-mediated activation of *XIAP*, although residual activity in the m1/m2 mutant suggests the involvement of additional regulatory elements. To identify the Zbtb38 domains critical for *XIAP* activation, we examined a series of deletion mutants. Luciferase assays showed that both full-length Zbtb38 and the ΔN-terminus (containing ZF1–5 but lacking the BTB domain) activated the 4.7 kb region of *XIAP*, whereas the ΔZF1–5 mutant (lacking ZF1–5) and the C-terminus (lacking both the BTB domain and ZF1–5) nearly lost this activity (Figure 3E). These results demonstrate that ZF1–5 is essential for *XIAP* transcriptional activation and that ZF1–5, together with the C-terminus, is sufficient for this function. Supporting this conclusion, immunoblotting showed that full-length Zbtb38 upregulated XIAP expression and suppressed apoptosis induced by serum deprivation, whereas the ΔZF1–5 mutant, despite being expressed at higher levels, failed to do so (Supplementary Figure S5). Collectively, these findings confirm that ZF1–5 is critical for Zbtb38-mediated *XIAP* activation and its anti-apoptotic function.

### Loss of Zbtb38 downregulates XIAP and Bcl-2, leading to increased apoptosis during ES cell differentiation and embryogenesis

**Figure 4:**
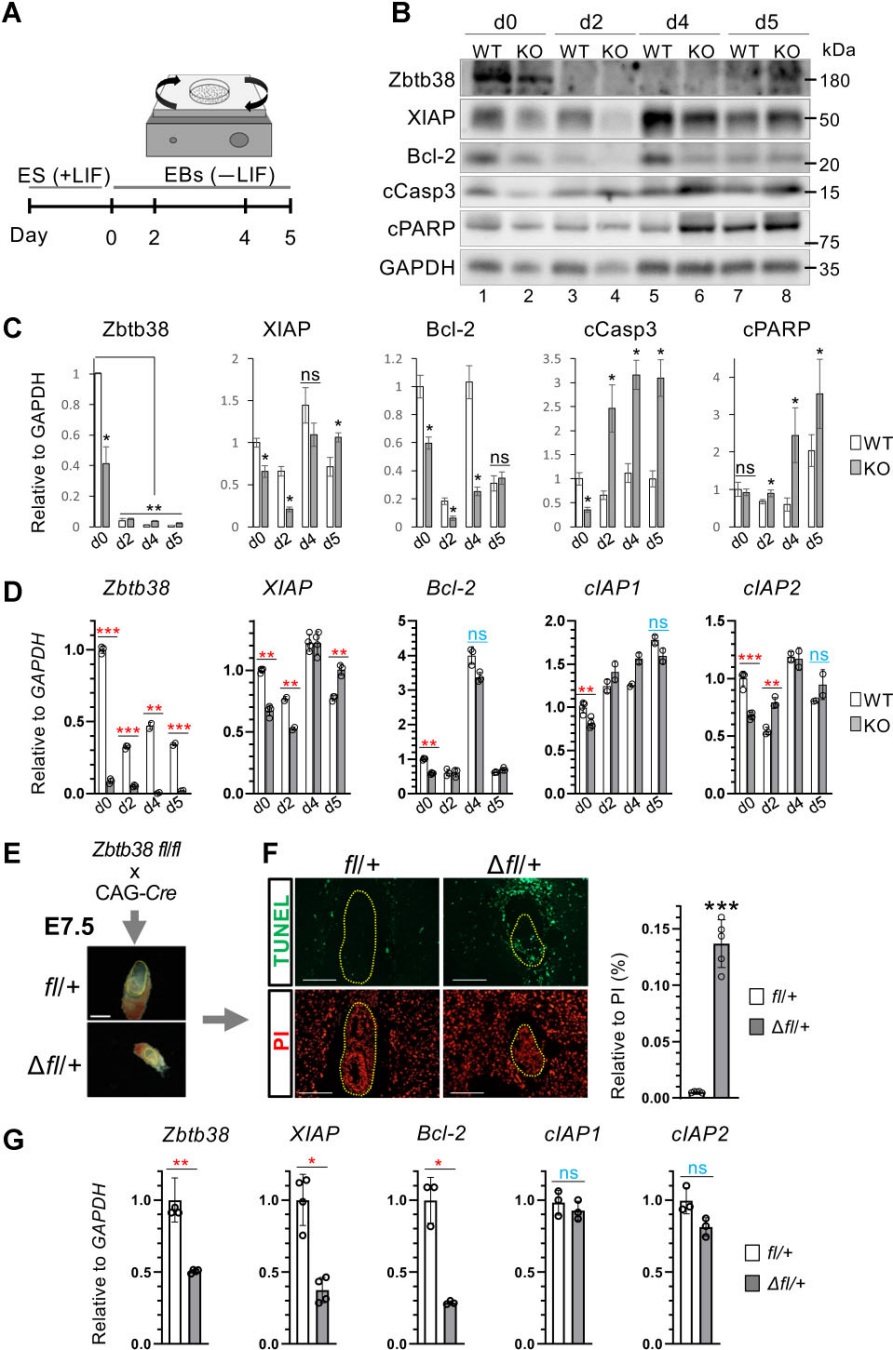
Zbtb38 loss downregulates *XIAP* and *Bcl-2*, increasing apoptosis during ES cell differentiation and embryogenesis. (**A**) Schematic of the ES cell differentiation assay. ES cells were cultured in ES medium (undifferentiated, d0) and then differentiated into embryoid bodies (EBs) under suspension culture without LIF, since LIF maintains mouse ES cells in an undifferentiated, pluripotent state. (**B** and **C**) Immunoblotting of proteins in wild-type (WT) and *Zbtb38* KO ES cells at the indicated differentiation days. Protein levels were quantified relative to GAPDH and normalized to WT at d0. (**D**) RT-qPCR analysis of gene expression in WT and *Zbtb38* KO ES cells during differentiation. Transcript levels were normalized to *GAPDH*. (**E**) Schematic of the strategy for generating *fl*/+ and ∆*fl*/+ embryos, with representative images of E7.5 embryos. fl, floxed allele. Scale bar, 100 μm. (**F**) TUNEL assay of paraffin-embedded E7.5 embryo sections. Quantification of TUNEL-positive cells relative to PI-counterstained nuclei is shown. *fl*/+ (*n* = 5), Δ*fl*/+ (*n* = 5). (**G**) RT-qPCR analysis of *Zbtb38* and apoptosis marker genes in E7.5 embryos. Transcript levels were normalized to *GAPDH. fl*/+ (*n* = 3), ∆*fl*/+ (*n* = 3). *P < 0.05, **P < 0.01, ***P < 0.001. ‘ns’ indicates no significance.

We previously reported that *Zbtb38* depletion (via knockdown or knockout) in undifferentiated ES cells does not induce apoptosis but instead reduces proliferation, suggesting increased apoptotic susceptibility upon differentiation (Nishii et al., 2012). To test this hypothesis, we induced differentiation of ES cells *in vitro* using embryoid body formation (Figure 4A). RT-qPCR analysis confirmed successful differentiation, as evidenced by the upregulation of ectodermal (*Sox2* and *Nestin*), endodermal (*Gata4* and *Gata6*), and mesodermal (*Brachyury* and *Mesp1*) markers from Day 0 (d0, undifferentiated) to d5 (Supplementary Figure S6). Immunoblotting showed progressive reductions in XIAP (d0–d2) and Bcl-2 (d0–d4) protein levels in *Zbtb38*-depleted cells, coinciding with increased apoptosis from d2 to d5 (Figure 4B and C). Notably, within the differentiation time course, XIAP and apoptotic markers do not necessarily change in a strictly inverse manner, indicating that differentiation-driven pathways also contribute to the regulation of XIAP and apoptosis markers in a Zbtb38-independent manner. RT-qPCR revealed decreased *XIAP* mRNA levels at d0–d2 and decreased *Bcl-2* mRNA levels at d0 in *Zbtb38* KO ES cells compared to controls (Figure 4D). Additionally, expression of *cIAP1, cIAP2*, and *Bcl-w* was also reduced in undifferentiated *Zbtb38* KO ES cells, whereas the expression of other apoptosis-related genes remained largely unchanged (Figure 4D; Supplementary Figure S7). These results suggest that Zbtb38 loss sensitizes ES cells to apoptosis during differentiation by downregulating *XIAP* and *Bcl-2*.

To investigate whether this mechanism functions *in vivo*, we focused on E7.5 embryos, because our previous data indicated that increased apoptosis occurs in *Zbtb38* heterozygous mutants at this stage (Nishio et al., 2022). Paraffin-embedded sections from Δ*flox*/+ (Δ*fl*/+) embryos, generated by intercrossing *Zbtb38 fl*/*fl* with CAG-*Cre* mice, were analyzed using the terminal deoxynucleotidyl transferase (TdT)-mediated dUTP nick-end labeling (TUNEL) assay to detect DNA fragmentation, a hallmark of apoptosis (Figure 4E). The results showed that *Zbtb38* Δ*fl*/*+* embryos exhibited a significantly higher number of TUNEL-positive nuclei compared to controls, correlating with their reduced size (Figure 4F). RT-qPCR further revealed that *XIAP* and *Bcl-2* mRNA levels decreased in Δ*fl*/+ embryos, while *cIAP1* and *cIAP2* expression remained unchanged (Figure 4G). Collectively, these data suggest that Zbtb38 loss promotes apoptosis during embryogenesis by downregulating *XIAP* and *Bcl-2*.

### Zbtb38 is a potential oncogenic driver and its knockdown triggers apoptosis in cancer cells

**Figure 5:**
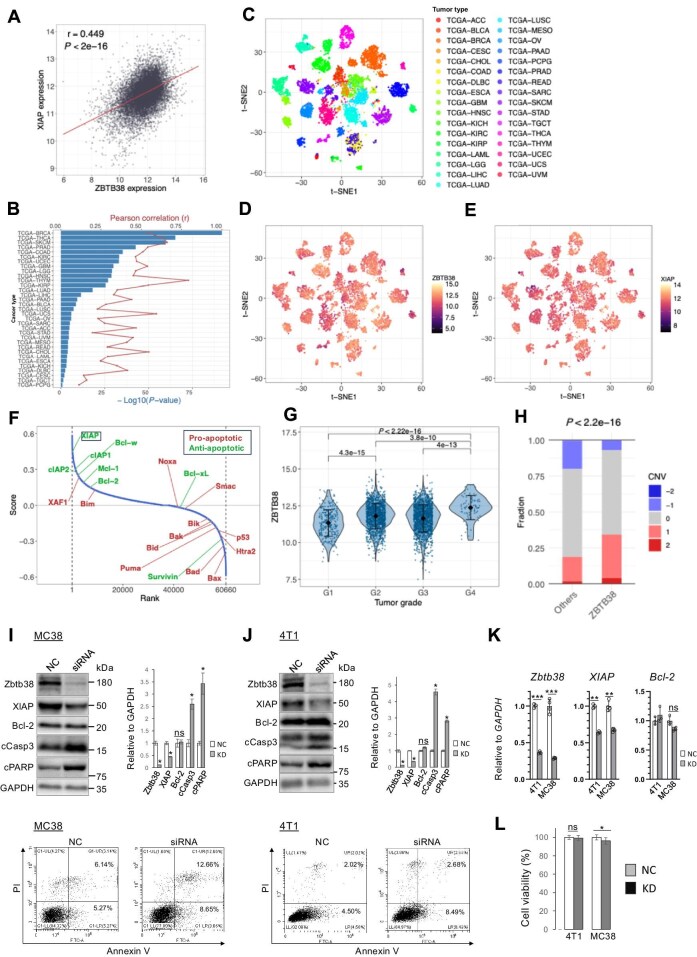
ZBTB38 is a potential oncogenic driver, and its knockdown induces apoptosis in cancer cells. (**A**) Scatter plot showing correlation between *ZBTB38* expression and *XIAP* expression across TCGA tumor types. Pearson’s correlation coefficients (r) and *P*-value are indicated. (**B**) Pearson’s correlation coefficients and −log10(*P*-value) values of the correlation between *XIAP* expression and *ZBTB38* expression across TCGA tumor types. (**C**) t-SNE projection of transcriptomic data from all TCGA tumor types. (**D** and **E**) Expression of *ZBTB38* (**D**) and *XIAP* (**E**) on t-SNE plots. (**F**) Gene ranking based on co-expression correlation with *ZBTB38*. Pro- and anti-apoptotic genes are labeled. (**G**) Distribution of *ZBTB38* mRNA expression levels across tumor grades (G1–G4) in all TCGA tumor types. (**H**) Fractions of CNV in high-grade tumors (G3 and G4) for all genes and for *ZBTB38. P*-value was calculated using a Chi-square test (df = 4). (**I**–**L**) MC38 and 4T1 cells were transfected with negative control siRNA (NC) or *Zbtb38* siRNA (KD). (**I** and **J**) Upper: immunoblotting of the indicated proteins with GAPDH serving as a loading control. Densitometric quantification (normalized to GAPDH) is shown. Lower: Annexin V–FITC/PI staining analyzed by flow cytometry. Percentages of early apoptotic cells (Annexin V^+^/PI^−^) and late apoptotic/necrotic cells (Annexin V^+^/PI^+^) are shown in the lower-right and upper-right quadrants, respectively. (**K**) RT-qPCR analysis of gene expression normalized to *GAPDH*. (**L**) Effects of *Zbtb38* knockdown on the viability of 4T1 and MC38 cells assessed by CCK-8 assay (*n* = 3). *P < 0.05, **P < 0.01, ***P < 0.001. ‘ns’ indicates no significance.

Our results demonstrate that Zbtb38 directly suppresses apoptosis by activating *XIAP* transcription, suggesting a potential role for Zbtb38 in cellular transformation and cancer progression. To further clarify this role, we analyzed gene expression data from The Cancer Genome Atlas (TCGA). Across all TCGA tumor types, *ZBTB38* expression exhibited a strong positive correlation with *XIAP* expression (Figure 5A). To further explore this relationship at the level of individual tumor samples, we performed t-distributed stochastic neighbor embedding (t-SNE) visualization using transcriptome data from all TCGA samples. Although samples clearly clustered by cancer type (Figure 5C), expression levels of *XIAP* and *ZBTB38* remained consistently positively correlated within each cancer-specific cluster, indicating that their co-expression is preserved across diverse tumor lineages (Figure 5D and E). Among the cancer types analyzed, particularly strong correlations were observed in cutaneous melanoma, thyroid carcinoma, prostate adenocarcinoma, breast carcinoma, and colon adenocarcinoma (Figure 5B). These trends were further validated using independent microarray datasets from the MERAV database (Supplementary Figure S8A). To extend these findings beyond human tumors, we analyzed the Cancer Cell Line Encyclopedia (CCLE). Consistent with the TCGA and MERAV results, ZBTB38 and XIAP expression showed a significant positive correlation (r = 0.303, *P* < 2 × 10⁻¹⁶) across a broad panel of human cancer cell lines (Supplementary Figure S8B). A genome-wide correlation analysis revealed that *ZBTB38* expression was negatively correlated with pro-apoptotic genes such as *Bax* and *Bad*, while showing positive correlations with anti-apoptotic genes, with *XIAP* displaying the strongest correlation (Figure 5F). Together, these findings indicate that ZBTB38 functions as a negative regulator of apoptosis, thereby promoting cancer cell survival and contributing to malignancy. To better understand the role of ZBTB38 in cancer progression, we also examined its expression across tumor grades. Higher *ZBTB38* expression was significantly associated with higher tumor grade (Figure 5G). Moreover, copy number variation (CNV) analysis revealed that *ZBTB38* copy number gain occurred more frequently in high-grade tumors (G3/G4) compared with the background CNV distribution (Figure 5H). These results support ZBTB38 as a potential oncogenic factor and a promising therapeutic target for promoting apoptosis in cancer cells.

To directly assess whether Zbtb38 loss induces apoptosis in cancer cells, we performed knockdown experiments in both *p53*-proficient MC38 (murine colon adenocarcinoma) and *p53*-deficient 4T1 (murine stage IV mammary carcinoma) cell lines. Immunoblotting revealed that *Zbtb38* knockdown led to decreased XIAP protein levels and increased levels of apoptotic markers in both cell lines, while Bcl-2 levels remained largely unchanged (Figure 5I and J). Consistent with these molecular changes, Annexin V/PI flow cytometry showed increased apoptotic populations, including an elevated Annexin V^+^/PI^−^ early apoptotic fraction (Figure 5I and J). RT-qPCR further confirmed the decreased *XIAP* mRNA expression following *Zbtb38* knockdown, with increased *Bak* expression, while *Bcl-2, p53*, and other apoptosis-related genes remained minimally affected (Figure 5K; Supplementary Figure S9). To evaluate the impact on overall cell viability, we performed CCK-8 assays in parallel. *Zbtb38* knockdown resulted in only a modest reduction in apparent viability in MC38 cells (∼96% of control) and no significant change in 4T1 cells (Figure 5L). This is consistent with CCK-8 primarily reporting total cellular metabolic activity, which can be less sensitive than Annexin V/PI flow cytometry to early apoptotic changes over this time frame. These results suggest that *Zbtb38* knockdown in cancer cells induces apoptosis primarily through *XIAP* downregulation, independently of Bcl-2 or p53 expression status.

**Figure 6:**
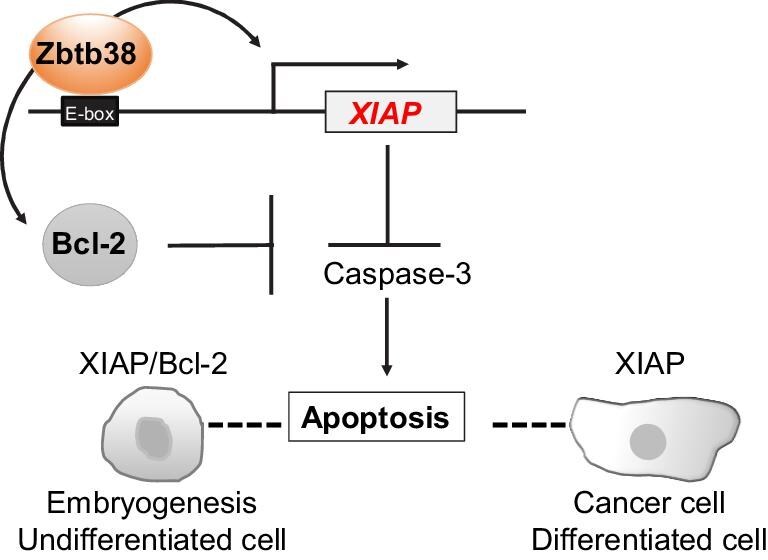
Schematic model of the Zbtb38–XIAP and Zbtb38–XIAP/Bcl-2 axis in apoptosis regulation. Zbtb38 activates *XIAP* transcription by binding to E-box motifs within the upstream regulatory region of the *XIAP* gene, thereby inhibiting the activation of caspases and suppressing apoptosis in cancer cells and differentiated cells. During embryogenesis and in undifferentiated cells, both *XIAP* and *Bcl-2*—transcriptional targets of Zbtb38—cooperatively inhibit caspase-mediated apoptosis and promote cell survival during development.

In conclusion, this study demonstrates that Zbtb38 regulates apoptosis by transcriptionally activating *XIAP* via E-box motifs in its upstream region, independently of p53 and DNA methylation, across various cell types, including cancer cells. During ES cell differentiation and embryogenesis, loss of Zbtb38 increases apoptotic sensitivity by downregulating both *XIAP* and *Bcl-2* (Figure 6). Furthermore, ZBTB38 expression correlates strongly with *XIAP* levels and is elevated in high-grade malignancies. These findings deepen the mechanistic understanding of apoptotic regulation and highlight Zbtb38 as a potential therapeutic target in cancers with dysregulated apoptosis.

## Discussion

This study identifies Zbtb38 as a novel positive regulator of *XIAP* that functions independently of p53 expression and DNA methylation. XIAP overexpression rescues apoptosis in *Zbtb38*-knockdown cells, supporting *XIAP* as a key downstream target, consistent with its established role in caspase inhibition. This axis appears broadly conserved in humans, as *ZBTB38* and *XIAP* show a robust positive correlation across TCGA tumors, and this relationship is independently supported by additional tumor expression datasets (MERAV) and human cancer cell lines (CCLE).

The anti-apoptotic function of Zbtb38 appears to be cell type-dependent. In ES cells, loss of Zbtb38 induces apoptosis during differentiation, accompanied by downregulation of XIAP and Bcl-2, suggesting that both proteins are essential for maintaining cell survival during lineage commitment. Notably, apoptotic-marker kinetics were context-dependent: acute somatic knockdown induces rapid cCasp3/cPARP, whereas ES-cell differentiation shows delayed cPARP accumulation after cCasp3 activation. The temporal sequence, characterized by *XIAP* and *Bcl-2* downregulation preceding apoptosis, supports a ‘priming’ model, in which undifferentiated ES cells tolerate Zbtb38 loss but become sensitized to apoptosis upon differentiation. This model is further supported by observations in E7.5 *Zbtb38* Δ*fl*/+ embryos, which exhibit increased apoptosis and decreased *XIAP* and *Bcl-2* expression. In contrast, in MEFs and cancer cell lines (e.g. 4T1 and MC38), *Bcl-2* levels remain unchanged following *Zbtb38* knockdown, suggesting that Bcl-2 is dispensable in these contexts, possibly due to intrinsic cell-type differences or oncogenic reprogramming. Additionally, the differential regulation of pro-apoptotic genes such as *Bik* and *Noxa* in *p53*-deficient cells suggests that Zbtb38 also regulates apoptosis via additional pathways, requiring further investigation.

Mechanistically, our ChIP and luciferase assays demonstrate that Zbtb38 binds to conserved E-box motifs within upstream regulatory regions of the *XIAP* gene and activates transcription through its DNA-binding region (ZF1–5) together with an adjacent activation region. Notably, unlike prior studies in which Zbtb38 was characterized as a transcriptional repressor using tethering assays (Sasai et al., 2005), our results indicate that Zbtb38 can function as a context-dependent transcriptional activator at the XIAP regulatory region. Although we did not perform electrophoretic mobility shift assay (EMSA) experiments in the present study, Kiefer et al. (2005) demonstrated by EMSA that ZENON, the rat ortholog of Zbtb38 (sharing ∼97% amino acid sequence identity within ZF1–5), directly binds to E-box motifs to activate transcription. Together with our ChIP–qPCR enrichment at conserved E-box regions and E-box mutagenesis/luciferase assays (Figure 3), these findings support direct, sequence-specific DNA binding in our system.

Although Zbtb38 binds to E-box motifs located ∼4.1–4.7 kb upstream of the *XIAP* TSS, clear evidence of typical enhancer marks such as p300 binding and H3K27ac enrichment in this region was not observed in publicly available ChIP-seq datasets from ES cells and other cell types. This absence suggests that activation of this region may be context-dependent or transiently induced during specific developmental stages or stress conditions that promote apoptosis. Interestingly, an E-box motif has also been identified in the *Bcl-2* promoter, although whether Zbtb38 regulates *Bcl-2* through this element remains unclear and requires further study (McGill et al., 2002). E-box motifs are canonical binding sites for basic helix–loop–helix (bHLH) transcription factors and are critical for regulating gene expression in diverse biological contexts, including development, stress responses, and disease (Pan et al., 2023). Zbtb38’s ability to activate transcription via E-box motifs expands its functional repertoire beyond methyl-CpG binding, distinguishing it from close homologs such as Zbtb33 (Kaiso) and Zbtb4, which bind to methylated DNA or non-methylated Kaiso-binding sequences but not E-box motifs (Buck-Koehntop et al., 2012; Buck-Koehntop and Defossez, 2013). Disrupting the Zbtb38–E-box interaction may therefore offer a novel therapeutic approach to suppress *XIAP* and promote apoptosis in tumors resistant to current *XIAP*-targeted therapies. Furthermore, genome-wide association studies have linked *ZBTB38* polymorphisms (e.g. rs6763931, which correlates with its expression in blood) to a variety of physiological and pathological traits, including human height, cancer susceptibility, and neurodegenerative disorders (Gudbjartsson et al., 2008; Kote-Jarai et al., 2011; Mead et al., 2012; Jing et al., 2019; Chen et al., 2019). These associations highlight the broad physiological and pathological significance of Zbtb38-mediated gene regulation, potentially through its ability to bind to E-box motifs.

XIAP is frequently overexpressed in various cancers and is associated with tumor progression, poor prognosis, and apoptosis resistance (Gao et al., 2019). Therapeutic approaches targeting *XIAP*, such as small-molecule inhibitors and antisense oligonucleotides, have shown promise in preclinical studies (Holcik et al., 2001). In contrast, the role of Zbtb38 in cancer remains much less defined and appears to be context-dependent. For example, ZBTB38 acts as a tumor suppressor in prostate cancer, where its loss correlates with tumor progression, but functions as an oncogene in bladder cancer, promoting migration, invasion, and survival (Jing et al., 2019; de Dieuleveult et al., 2020). In this study, TCGA analysis reveals a strong correlation between *ZBTB38* and *XIAP* expression across multiple tumor types, with *ZBTB38* expression elevated in high-grade malignancies. Consistently, *Zbtb38* knockdown induced apoptosis in both *p53*-proficient (MC38) and *p53*-deficient (4T1) cancer cells, leading to reduced *XIAP* expression, while *Bcl-2* levels remained unchanged. These findings support the conclusion that Zbtb38 mediates apoptosis primarily through *XIAP* regulation, independently of p53. However, validation in additional cancer cell lines from diverse tumor types is necessary to confirm the generalizability of these findings. This is particularly important given that *p53* is mutated or inactivated in ∼50% of human cancers, contributing to therapeutic resistance. Therefore, targeting the *Zbtb38–XIAP* axis may offer a promising therapeutic strategy, particularly for *p53*-deficient tumors where *p53*-dependent apoptotic pathways are compromised.

In summary, this study demonstrates that Zbtb38 regulates apoptosis by modulating *XIAP* and, in some contexts, *Bcl-2* in a cell type- and context-dependent manner. We show that this function is independent of p53 and DNA methylation, providing new insights into the roles of Zbtb38 in both differentiated and undifferentiated cells, including cancer cells. These findings deepen our mechanistic understanding of apoptotic regulation and highlight Zbtb38 as a potential therapeutic target in *XIAP*-driven and *p53*-deficient cancers. Future studies exploring Zbtb38’s broader transcriptional network and context-specific interactions will be essential to further elucidate its physiological and pathological functions.

## Materials and methods

### Mice

*Zbtb38* ∆*fl*/+ mice were generated by randomly crossing 6- to 8-week-old female *Zbtb38 fl*/*fl* mice with 8- to 16-week-old CAG-*Cre* mice, and *vice versa*. Embryos of both sexes were used due to the breeding scheme; no sex-specific effects were observed, and data were pooled. Genotyping was performed as previously described (Nishio et al., 2022). CAG-*Cre* mice were kindly provided by Dr Masaru Okabe (Osaka University). Wild-type C57BL/6J mice were obtained from CLEA. All animal experiments were approved by the Nara Institute of Science and Technology Animal Care Committee and were conducted in accordance with Japanese guidelines.

### Cell culture and ES cell differentiation assay

C2C12 cells were cultured in Dulbecco’s modified Eagle’s medium (DMEM; Nacalai Tesque) supplemented with 15% fetal bovine serum (FBS; Sigma-Aldrich), 2 mM L-glutamine (Nacalai Tesque), and 1% penicillin/streptomycin (Nacalai Tesque) as previously described (Oikawa et al., 2011). *p53* KO and *p53*/*Dnmt1* DKO MEFs were kindly provided by Dr Richard Meehan (MRC Human Genetics Unit, University of Edinburgh). Immortalized MEFs were established in-house by stable transfection of primary C57BL/6 MEFs with SV40 large T antigen. MEFs, *p53* KO MEFs, and *p53*/*Dnmt1* DKO MEFs were maintained in the same medium, supplemented with 10% FBS and 1 mM sodium pyruvate (Invitrogen). NMuMG, 4T1, and HEK293T cells were cultured in DMEM with 10% FBS, 2 mM L-glutamine, and 1% penicillin/streptomycin. MC38 cells were maintained in the same medium with the addition of 100 μM non-essential amino acids and 1 mM sodium pyruvate.

Mouse RF8 (wild-type) and *Zbtb38* KO ES cells were cultured as previously described (Nishii et al., 2012). Briefly, undifferentiated ES cells were grown on mitomycin C-treated SNL-STO feeder cells in DMEM containing 15% FBS, 2 mM L-glutamine, 100 μM non-essential amino acids, 1% penicillin/streptomycin, 0.1 μM β-mercaptoethanol, 1000 IU/ml leukemia inhibitory factor (LIF), and 3i inhibitors (3 μM CHIR99021, 1 μM PD0325901, and 10 μM SB431542; Selleck Chemicals). Differentiation was induced by culturing cells in the same medium without LIF and 3i, allowing embryoid body formation in suspension, as previously described (Kotoku et al., 2016). Mouse ES cells were tested for mycoplasma contamination prior to use, and all cultured cells exhibited normal growth and morphology.

### Reporter constructs and luciferase assays

To generate the pGL3-XIAP 4.7 kb construct, genomic DNA from C2C12 cells was PCR-amplified using primers containing *Kpn*I and *Xho*I sites (Supplementary Table S1), and the fragment was cloned into the similarly digested pGL3 vector. E-box mutant constructs (pGL3-XIAP m1 and pGL3-XIAP m1/m2) were generated by site-directed mutagenesis (Takara Bio) following the manufacturer’s protocol. All constructs were sequence-verified using an ABI PRISM 3130 genetic analyzer. Primer sequences are listed in Supplementary Table S1. Plasmids (pcDNA3-2×Flag, pcDNA3-2×Flag-Zbtb38, pcDNA3-Myc, pcDNA3-Myc-Zbtb38, and pcDNA3-Myc-Zbtb38 ΔZF1–5) were prepared as previously described (Sasai et al., 2005). Luciferase assays were performed as described previously (Kotoku et al., 2016). Briefly, HEK293T cells were seeded in 24-well plates and transfected with 0–250 ng of expression plasmids and 100 ng of firefly luciferase reporter (pGL3 or pGL3-XIAP 4.7 kb), along with 4 ng of pRL-TK (Renilla luciferase) as an internal control. Total DNA amounts were adjusted with empty pcDNA3. Luciferase activity was measured 32–48 h post-transfection using the Dual-Luciferase Reporter Assay System (Promega), and firefly luciferase values were normalized to Renilla activity. Assays were conducted in duplicate across three independent experiments.

### siRNA and transient transfection

Cells were transfected with 5–10 nM *Zbtb38*-specific Dicer substrate siRNA duplex or a scrambled negative control (Integrated DNA Technologies) using Lipofectamine RNAiMAX (Thermo Fisher Scientific), as previously described (Oikawa et al., 2008). Downstream assays, including semi-quantitative PCR, RT-qPCR, and immunoblotting, Annexin V/PI flow cytometry, and cell viability assays, were performed 24–48 h post-transfection. Plasmid transfection was performed using Lipofectamine 2000 (Invitrogen), PEI-Max (Polysciences), or ViaFect (Promega), according to the manufacturers’ protocols. Cells were harvested 24–48 h post-transfection. For rescue experiments, C2C12 cells or p53 KO MEFs were transfected with either an empty vector (pcDNA3-2×Flag) or an XIAP expression plasmid (untagged pcDNA3-XIAP or pcDNA3-2×Flag-XIAP) for 24 h, followed by transfection with scrambled negative control or *Zbtb38* siRNA for an additional 24 h.

### Annexin V/PI flow cytometry

Cells were transfected with 10 nM *Zbtb38*-specific Dicer-substrate siRNA duplexes or a scrambled negative control. Cells were harvested 36–48 h post-transfection, washed with cold phosphate-buffered saline (PBS), and stained with Annexin V–FITC and PI in binding buffer according to the manufacturer’s instructions (eBioscience™ Annexin V Apoptosis Detection Kit FITC, Cat. no. 88-8005-74). Samples were analyzed on a CytoFLEX flow cytometer (Beckman Coulter) and data were analyzed using CytExpert software. Early apoptotic cells were defined as Annexin V⁺/PI⁻ and late apoptotic/necrotic cells as Annexin V⁺/PI⁺.

### Cell viability assay

Cell viability was assessed using a WST-8 assay (CCK-8; Dojindo). Cells were transfected with 10 nM Zbtb38-specific Dicer-substrate siRNA duplexes or a scrambled negative control, plated into 96-well plates, and incubated for 36–48 h. WST-8 reagent was added according to the manufacturer’s instructions, and absorbance at 450 nm was measured using a TriStar² multimode microplate reader (Berthold). Background absorbance from blank wells was subtracted, and values were normalized to the negative control.

### RNA extraction, RT-qPCR, and semi-quantitative PCR

Total RNA was extracted using Sepasol RNA Super G (Nacalai Tesque) for cultured cells or the PicoPure RNA Isolation Kit (Thermo Fisher Scientific) for E7.5 embryos. cDNA synthesis was performed using 500 ng (cultured cells) or ∼5 ng (embryos) of total RNA and ReverTra Ace qPCR RT Master Mix with gDNA Remover (TOYOBO). RT-qPCR was performed using Thunderbird SYBR Green PCR Mix (TOYOBO) on a LightCycler® 96 (Roche), with *GAPDH* as the internal control. Data were analyzed using the 2-delta-delta Ct method, as previously described (Kotoku et al., 2016). For semi-quantitative PCR, reactions were run at 58°C annealing temperature, with *GAPDH* as the internal control. PCR products were separated on 2% agarose gels and visualized by ethidium bromide staining. Primer sequences are provided in Supplementary Tables S2 and S3.

### Western blot analysis

Protein lysates were prepared in RIPA buffer with protease inhibitors (Roche Diagnostics) as previously described (Sasai et al., 2005). Equal amounts of protein were separated by 6%–15% SDS–PAGE and transferred to polyvinylidene fluoride membranes. Blots were probed with antibodies against Zbtb38 (Sasai et al., 2005), XIAP (MBL, M044-3), Bcl-2 (MBL, D038-3), cleaved caspase-3 (cCasp3; Cell Signaling Technology, #9664), cleaved PARP (cPARP; Cell Signaling Technology, #9542), and GAPDH (Proteintech, 60004-1-Ig). Expected molecular weights: Zbtb38 (175 kDa), XIAP (55 kDa), Bcl-2 (26 kDa), cCasp3 (17 kDa), cPARP (89 kDa), and GAPDH (36 kDa). Horseradish peroxidase-conjugated secondary antibodies (anti-mouse #7076 and anti-rabbit #7074) were from Cell Signaling Technology. Signals were detected using a FUSION chemiluminescence imaging system (Thermo Fisher Scientific).

### ChIP assay and ChIP–qPCR

ChIP assays were performed as previously described (Oikawa et al., 2008; Kotoku et al., 2016). Briefly, C2C12 or *p53* KO MEFs were cross-linked with 1% formaldehyde, quenched with 0.125 M glycine for 15–30 min. After washing with PBS, cells were lysed in SDS lysis buffer and sonicated on ice to obtain ∼500-bp DNA fragments. Lysates were pre-cleared with Protein G Sepharose beads and immunoprecipitated with anti-Zbtb38 antibody or normal rabbit IgG (negative control). Input DNA (2%) and immunoprecipitated DNA were reverse-crosslinked and analyzed by RT-qPCR using primers listed in Supplementary Table S4. ChIP signals were normalized to input DNA. All experiments were performed in triplicate.

### Embryo dissection and TUNEL assay

Morphological and histological analyses of embryos were performed on E7.5 embryos. Bright-field images were captured using a Nikon E800M inverted microscope. For histological analysis, embryos were rinsed with ice-cold PBS, fixed in 4% paraformaldehyde at room temperature for 10–20 min, dehydrated through graded alcohols, and embedded in paraffin. Paraffin sections were used for the TUNEL assay (MBL, #8445) as previously described (Nishio et al., 2022). Briefly, sections were deparaffinized, labeled with TdT and nucleotide mix, counterstained with 1 μg/ml PI, and mounted. Fluorescent images were acquired using a TCS SP8 confocal microscope (Leica Microsystems).

### Data acquisition and processing for human tumor data analysis

The transcriptome data of human tumors were downloaded from TCGA using the TCGA biolinks package (v2.30.4) in R (v4.3.2). Raw count data were processed with DESeq2 (v1.42.1), and a variance stabilizing transformation was applied prior to downstream analyses. For correlation analyses, Pearson’s correlation coefficients and *P*-values were calculated in R to assess the association between target gene expression levels. Principal component analysis and t-SNE were performed for visualization using the irlba package (v2.3.5.1) and Rtsne (v0.17), respectively. Gene expression data for human cancer cell lines were obtained from CCLE/DepMap. The CCLE correlation results shown in Supplementary Figure S8B are provided in Supplementary Table S5. Gene-level RNA expression values (log2[TPM + 1]) were used for analyses, and Pearson’s correlation coefficients and *P*-values were calculated across cell lines using the same procedure as for TCGA. For the analysis of microarray-based gene expression data, batch-adjusted raw expression values were obtained from the MERAV database (http://merav.wi.mit.edu). Tumor grades were retrieved from TCGA clinical metadata, and substage labels (e.g. ‘IIIA’ and “IIIB”) were consolidated into major stage groups (e.g. ‘III’) for clarity. Gene-level thresholded CNV data for all TCGA tumor types were downloaded from the UCSC Xena Browser (https://xenabrowser.net/). CNV calls in this dataset were based on the GISTIC2 algorithm and categorized into five discrete levels (−2: deep deletion; −1: shallow deletion; 0: diploid; 1: low-level gain; 2: high-level amplification). CNV scores were integrated with the clinical and expression data using custom R scripts. All custom analysis scripts are publicly available at https://github.com/Toshi-Shigeoka/Zbtb38_paper/.

### Quantification and statistical analysis

Statistical analyses for qPCR and immunoblotting were conducted using GraphPad Prism 10 and Microsoft Excel. Unless otherwise indicated, data are presented as mean ± standard deviation (SD) from three independent experiments. Comparisons between two groups were performed using unpaired two-tailed Student’s *t*-tests, and one-way ANOVA was used for comparisons among more than two groups. To minimize potential biases, animals were housed in randomized cage positions, and samples were processed in a non-sequential order. Statistical significance was defined as *P < 0.05, **P < 0.01, and ***P < 0.001; ‘ns’ indicates no significance (*P* > 0.05).

### Data availability

Any additional data are available from the corresponding author upon reasonable request. All analysis scripts and processed data used for statistical analysis and figure generation are publicly available in the following GitHub repository: https://github.com/Toshi-Shigeoka/Zbtb38_paper/.

## Supplementary Material

mjag011_Supplemental_Files

## Notes

**Author contributions:** T.S. planned the study, conducted the bioinformatic analysis, prepared the figures, analyzed the data, and provided technical guidance and advice. H.N. and S.T. conceived, designed, and performed the experiments and analyzed the data. E.M. conceived, designed, and performed the experiments, analyzed the data, prepared the figures, and drafted the manuscript. N.A.N., Y.B., and Y.I. supervised the study and approved the final manuscript.
