# Supplementary material for: Zbtb38 transcriptionally activates XIAP to regulate apoptosis in development and cancer
Source: J Mol Cell Biol. 2026 Mar 17;18:mjag011. doi: 10.1093/jmcb/mjag011 (PMC13344426; doi:10.1093/jmcb/mjag011)
Supplement: mjag011_Supplemental_Files [file mjag011_supplemental_files.zip › JMCB-2025-0566.R1_Supplementary material.pdf]

## S1A

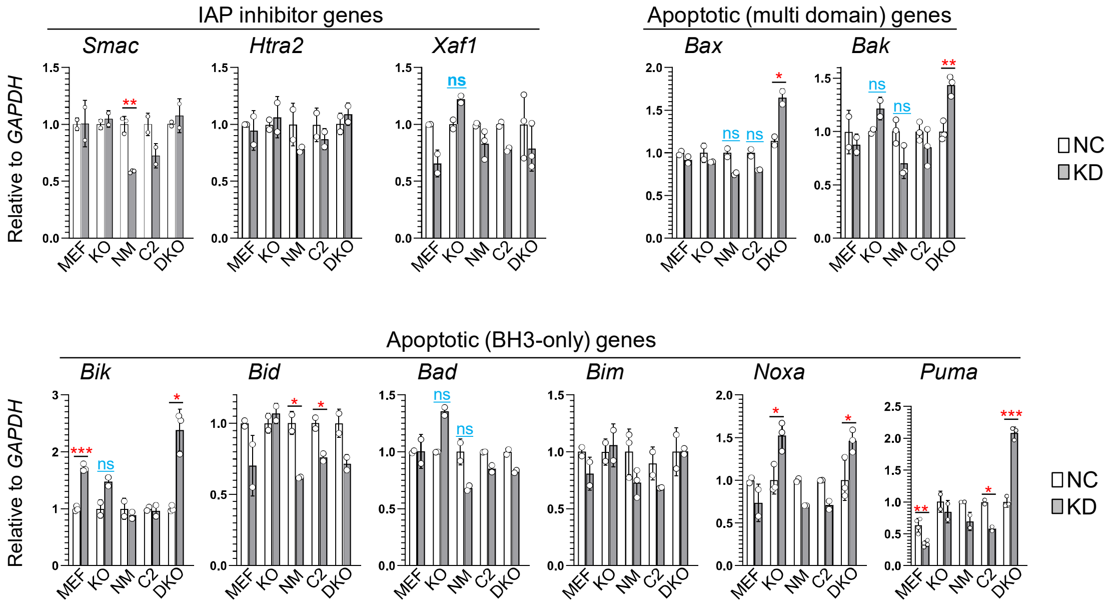


## S1B

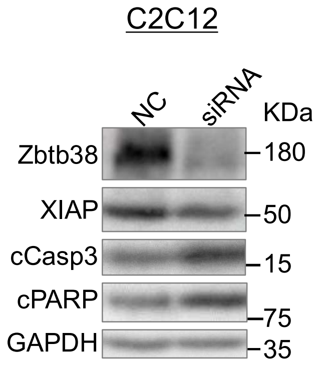


## S1C

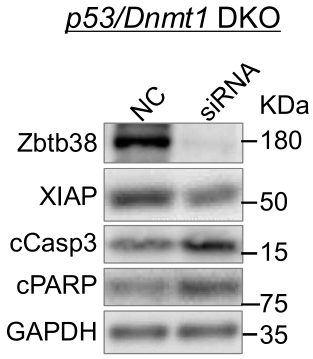


**Supplementary Figure S1** Expression of apoptosis-related genes following *Zbtb38* knockdown in *p53*-expressing (MEFs, NMuMG, C2C12) and *p53*-deficient (p53 KO, DKO) cell lines. **(S1A)** mRNA levels were quantified by RT-qPCR in negative control (NC, empty bars) and *Zbtb38* knockdown (KD, gray bars) groups, normalized to *GAPDH* (set to "1"). Abbreviations: KO, *p53* knockout MEFs; NM, NMuMG; C2, C2C12; DKO, *p53*/*Dnmt1* double-knockout MEFs. *$p < 0.05$, **$p < 0.01$, ***$p < 0.001$; ns – not significant. **(S1B and C)** *Zbtb38* was knockdown using siRNA in C2C12 cells (**S1B**) and *p53*/*Dnmt1* DKO MEFs (**S1C**), and protein expression was analyzed by immunoblotting 30–36 h post-transfection. NC – negative control; siRNA – *Zbtb38* siRNA duplex. GAPDH was used as a loading control.

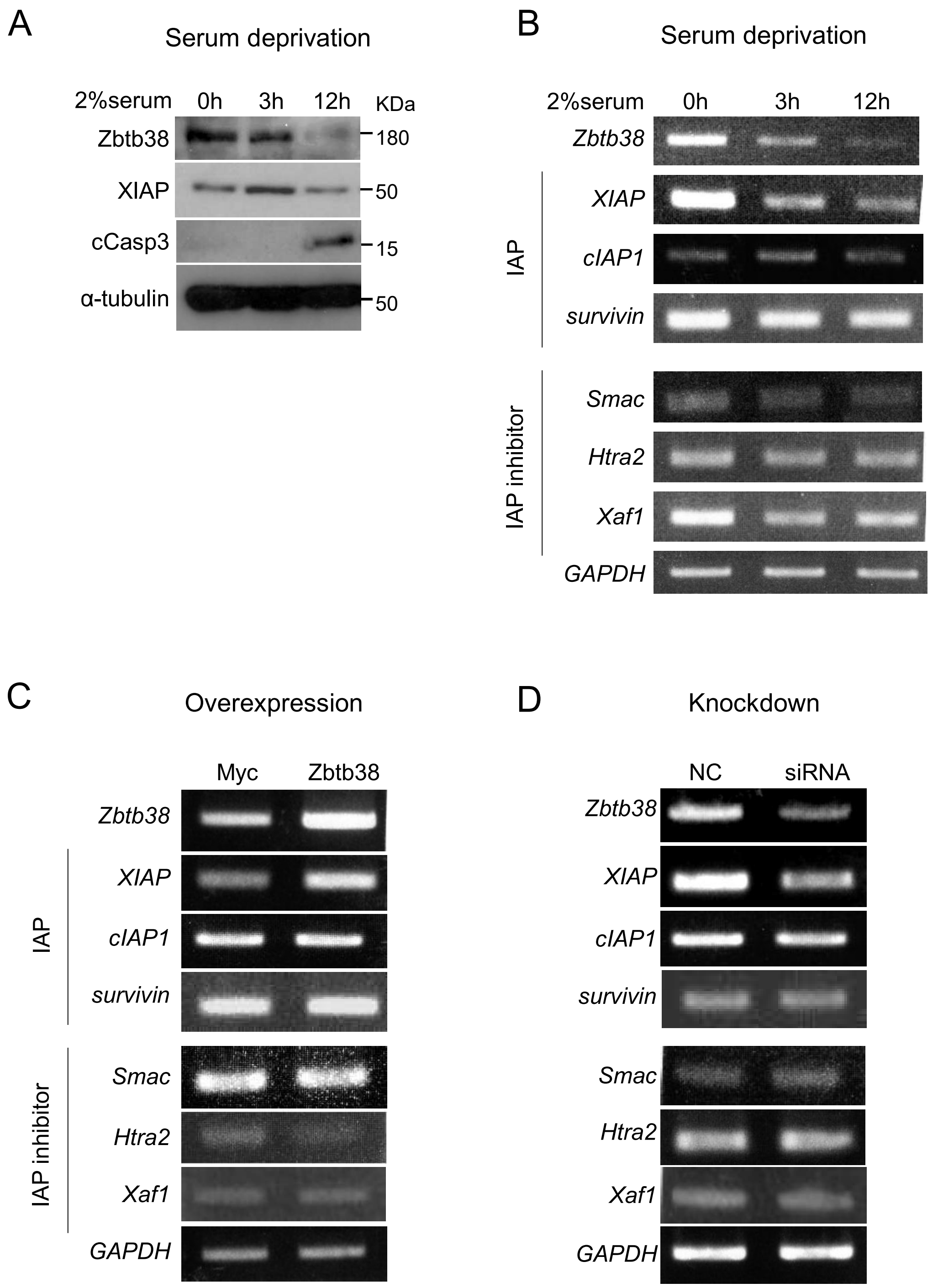


**Supplementary Figure S2**

Stress-associated downregulation of Zbtb38 and XIAP, and expression analysis of apoptosis-related genes after Zbtb38 overexpression or knockdown in C2C12 cells. **(A)** C2C12 cells were cultured under low-serum conditions (2% serum) for the indicated times (0, 3, and 12 h). Protein levels of Zbtb38, XIAP, and cCasp3 were assessed by immunoblotting; α-tubulin served as a loading control. **(B)** Under the same low-serum time course as in **(A)**, semi-quantitative RT-PCR was performed to analyze mRNA levels of the indicated IAP- and IAP inhibitor–related genes; *GAPDH* was used as an internal control. **(C, D)** Semi-quantitative RT-PCR analysis of the indicated genes after *Zbtb38* overexpression (C; Myc vs. Myc-Zbtb38, 48 h) or *Zbtb38* knockdown (D; negative control siRNA vs. *Zbtb38* siRNA, 36 h) in C2C12 cells. *GAPDH* was used as an internal control.

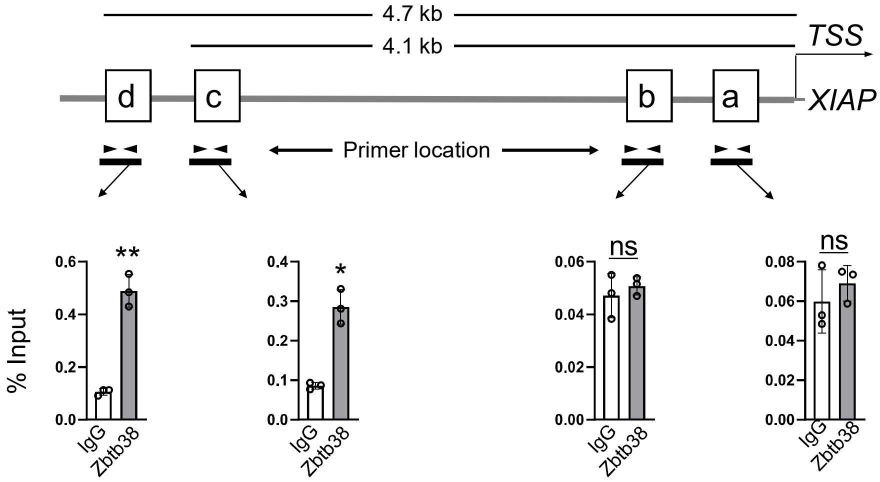


**Supplementary Figure S3** ChIP-qPCR results for *p53* KO MEFs. Schematic representation of the regulatory regions of *XIAP*. Regions a and b represent putative promoter regions, and regions c and d indicate the enhancer regions of *XIAP*. *TSS*, transcriptional start site. IgG-precipitated DNA (negative control) and Zbtb38-immunoprecipitated DNA were amplified by qRT-PCR using primers at the indicated locations. Data are shown as fold enrichment relative to the input control, which was set to 1. *$p < 0.05$, **$p < 0.01$. "ns" indicates no significance.

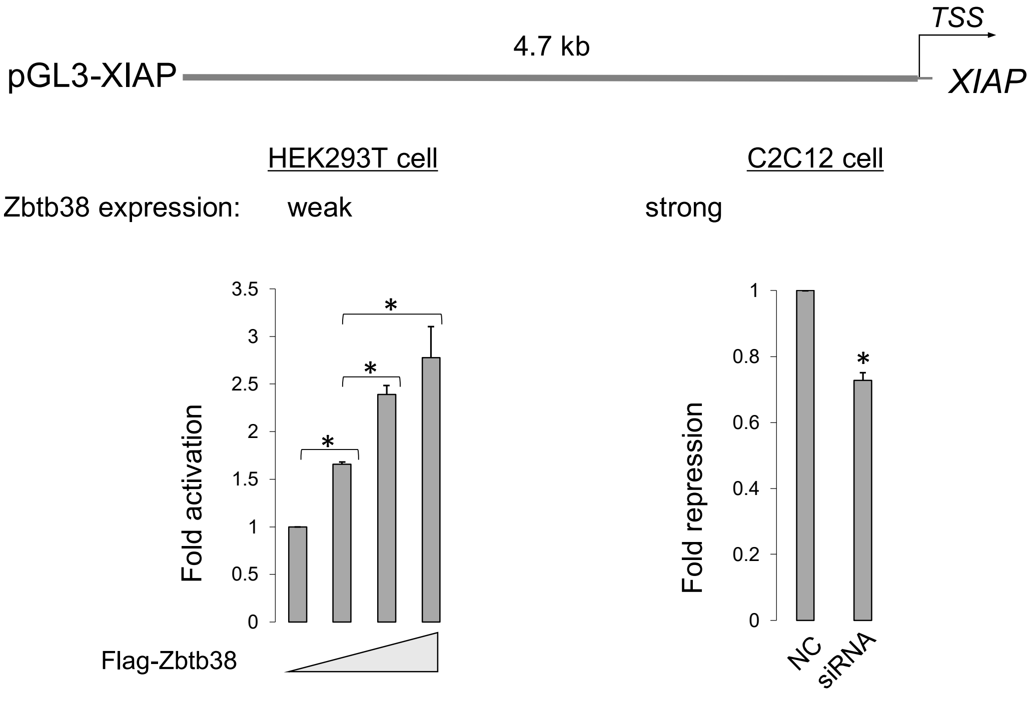


**Supplementary Figure S4** Zbtb38 overexpression in HEK293T cells and knockdown in C2C12 cells. Top panel: schematic of the pGL3-XIAP. Left panel: pGL3 and pGL3-XIAP were co-transfected with expression vectors for Flag or increasing amounts of Flag-Zbtb38 into HEK293T cells for 40-48 h. Luciferase activity was determined relative to that of Flag/pGL3, which was set to 1. Right panel: pGL3 vector and pGL3-XIAP were co-transfected with a negative control (NC) or siRNA against *Zbtb38* for 48 h. Luciferase activity was determined relative to that of pGL3, which was set to 1. *$p < 0.05$.

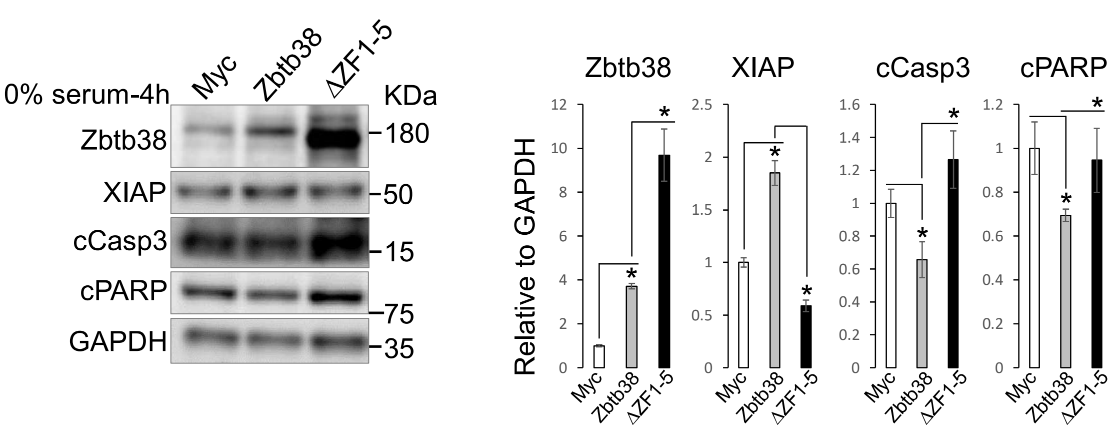


**Supplementary Figure S5** ZF1-5 of Zbtb38 is required for inhibiting apoptosis in C2C12 cells. Myc, Myc-Zbtb38, and Myc-ΔZF1-5 were transfected into C2C12 cells for 36 h and then switched to medium without FBS for 4 h. Data were quantified relative to GAPDH, which was set to 1, and are shown in the right panel. *$p < 0.05$.

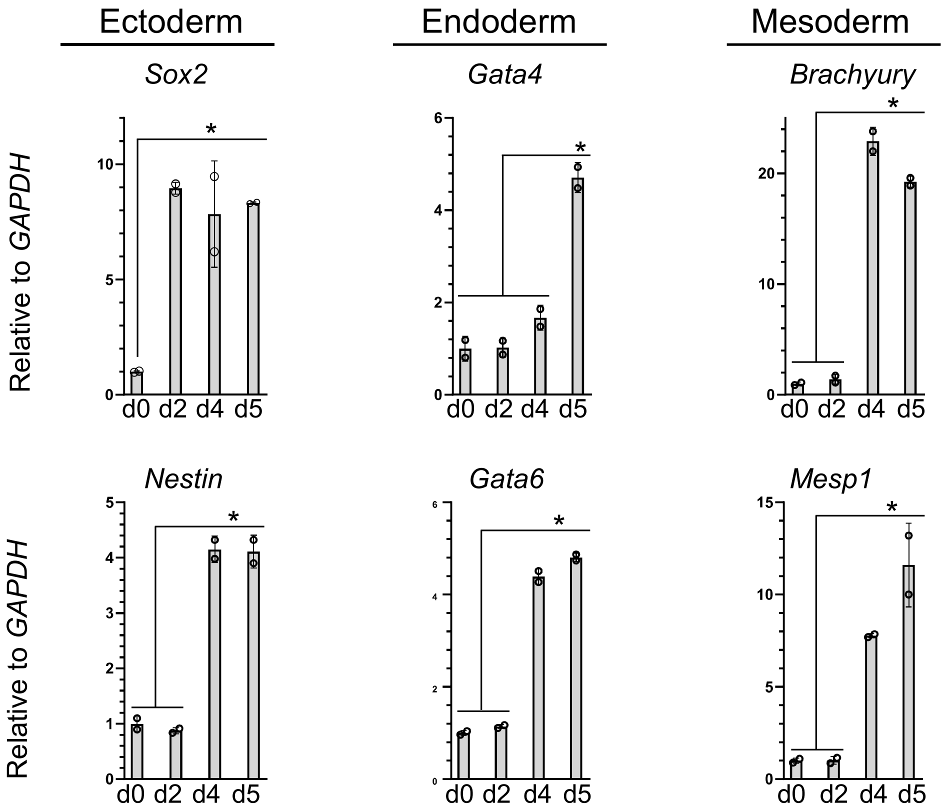


**Supplementary Figure S6** RT-qPCR results of the indicated gene expression in wild-type ES cells at the indicated differentiation days. ES cells were cultured in ES medium to maintain an undifferentiated state (d0) and then differentiated into embryoid bodies in suspension culture lacking leukemia inhibitory factor. The data are representative of three independent experiments. Transcript levels were normalized to those of *GAPDH*. $*p < 0.05$.

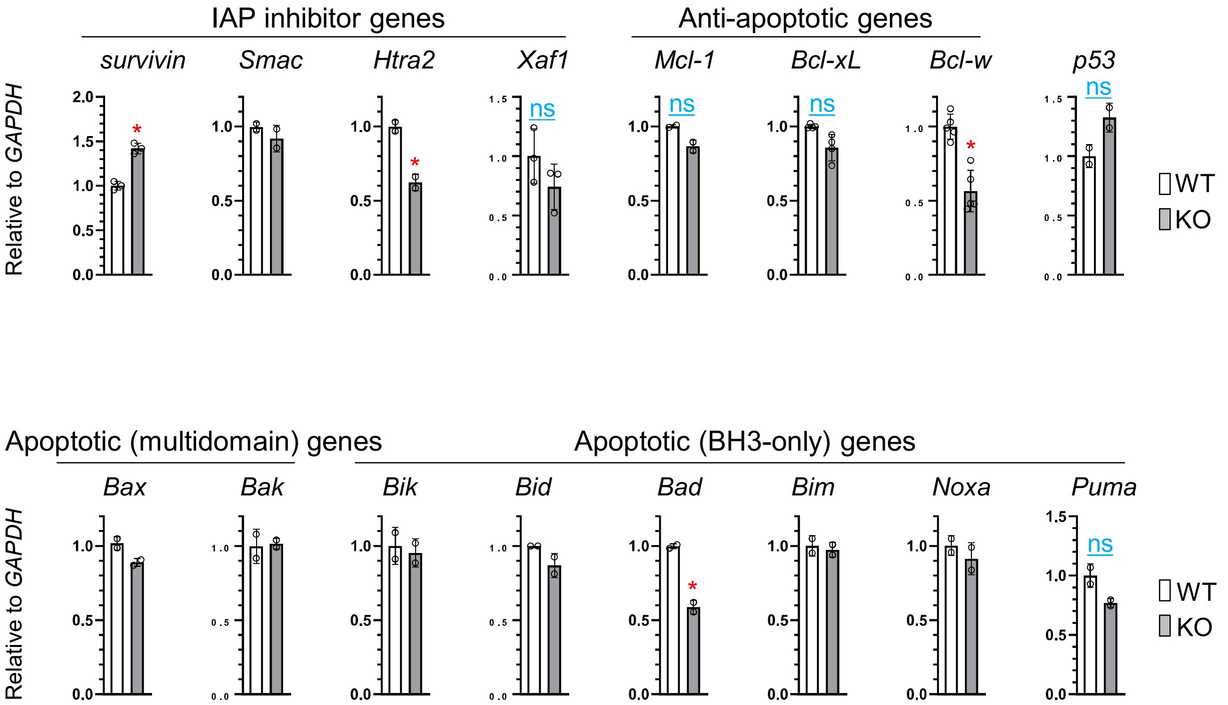


**Supplementary Figure S7** RT-qPCR analysis of the expression of indicated genes in undifferentiated ES cells. WT (empty bars) and KO (*Zbtb38* knockout, gray bars) ES cells were used. Each transcript was normalized to that of *GAPDH*, which was set to 1. *$p < 0.05$. "ns" indicates no significance.

Supplementary Fig. S8

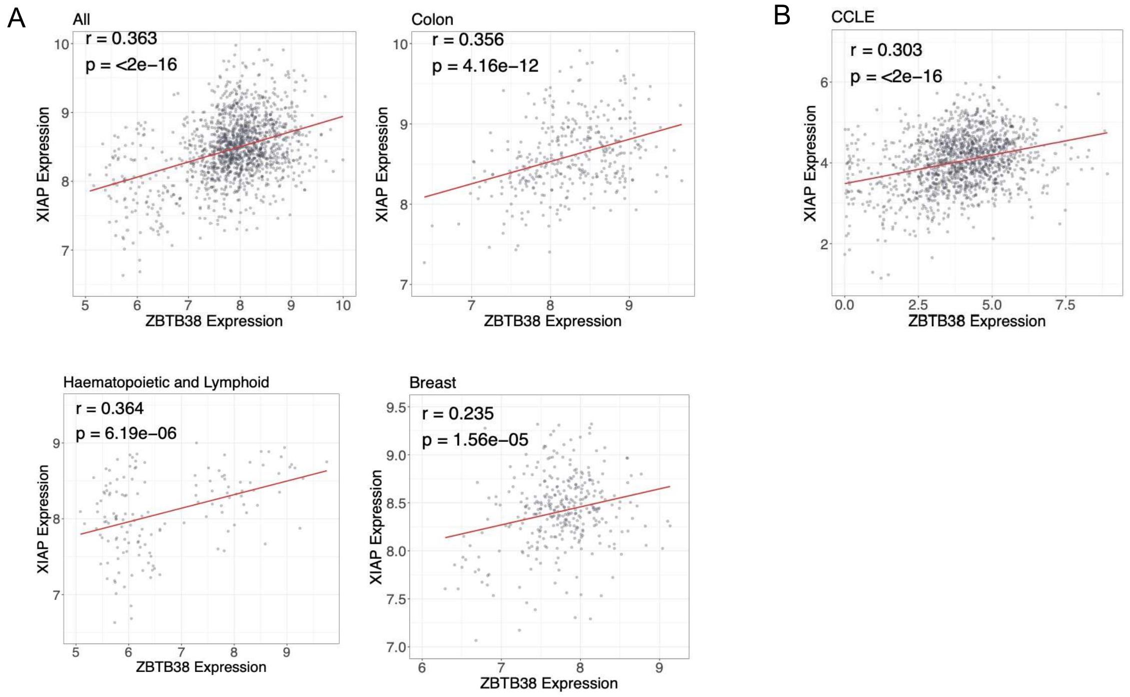


**Fig. S8 Expression analysis of ZBTB38 in human tumors and cancer cell lines using MERAV and CCLE.** Scatter plots show the correlation between ZBTB38 and XIAP expression in human tumors from the MERAV database (A) and in cancer cell lines from the CCLE database (B). Pearson's correlation coefficient (r) and p-values are indicated in each plot.

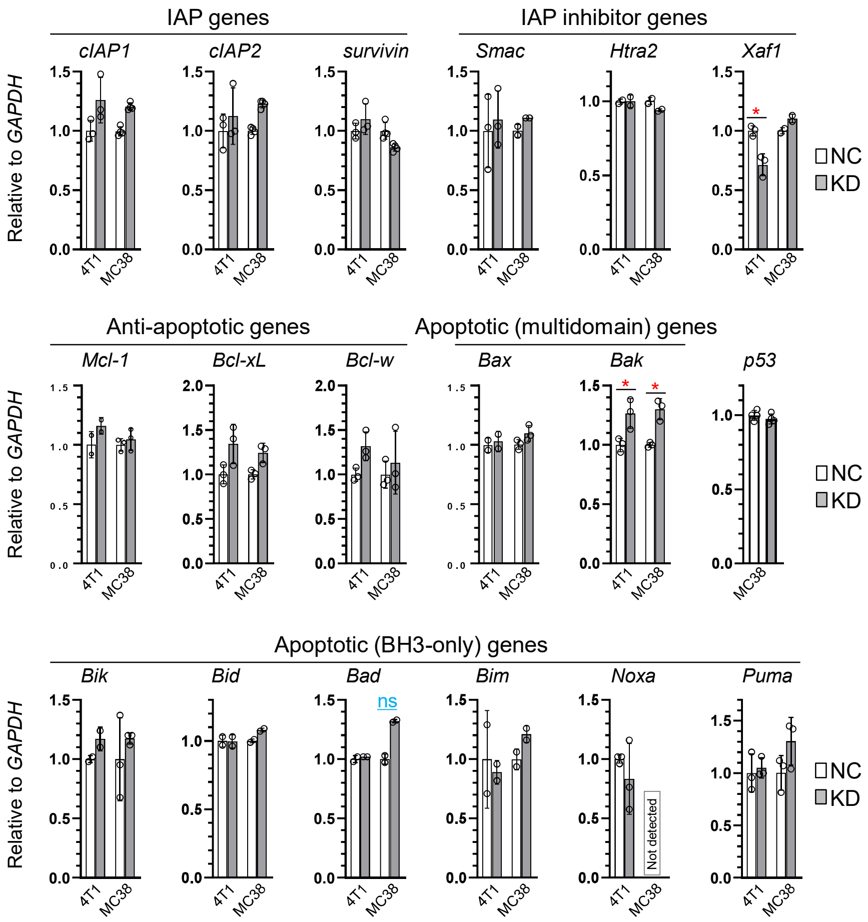


**Supplementary Figure S9** RT-qPCR analysis of gene expression in 4T1 and MC38 cells. WT (empty bars) and KO (*Zbtb38* knockout, gray bars) cells were used. Each transcript was normalized to *GAPDH*, set to 1. $p < 0.05$. "ns" indicates no significance.

**Supplementary Table S1 Primers used for the generation of reporter constructs.**

| Construct | Primer sequence |
|---|---|
| pGL3-XIAP 4.7 kb | F: CGGGGTACCCATTGGCCTTAATTCCACTG<br>R: CCGCTCGAGGGACTATTTCTGATACTAAGCAC |
| pGL3-XIAP m1 | TGAGTGTTCCAGTGCTGGACAGTAA<br>TTACTGTCCAGCACTGGAACACTCA |
| pGL3-XIAP m1/m2 | CAGAATGTTTTCAAAGTGCTGGAAAAATGACAG<br>GTGTCATTTTTCCAGCACTTTGAAAACATTCTG |

**Supplementary Table S2 Primer sequences for RT-qPCR.**

| Gene | Primer sequence (5′ to 3′) | Product size |
|---|---|---|
| *Zbtb38* | F: ACGCTCAAGATCCACGAGAG<br>R: ACCCTGAAAGCAGAACTGACA | 69 bp |
| *XIAP* | F: AACAAAGTGGGAAGGTGAGAG<br>R: TGTGTATGTGTCTGGGTGTAAG | 101 bp |
| *cIAP1* | F: ATTGGAAGAGCAGTTGCGG<br>R: CAGTCCCCTTGATTGTCCCC | 266 bp |
| *cIAP2* | F: CTGGCTATTTCAGTGGCTCTTA<br>R: TGCAAAGTGGTAGGGACTTG | 105 bp |
| *Bcl-2* | F: CTTCGCAGAGATGTCCAGTC<br>R: AGGGCGATGTTGTCCACCAG | 192 bp |
| *Bax* | F: ATGCGTCCACCAAGAAGCTGAG<br>R: CCCCAGTTGAAGTTGCCATCAG | 166 bp |
| *Bcl-xL* | F: GTGAAGCAAGCGCTGAGAG<br>R: AACTTGCAATCCGACTCACC | 250 bp |
| *Noxa* | F: AGTTCGCAGCTCAACTCAGG<br>R: GCCGTAAATTCACTTTGTCTCC | 201 bp |
| *Puma* | F: AGACAAGAAGAGCAGCATCG<br>R: CTAGTTGGGCTCCATTTCTGG | 117 bp |
| *Bik* | F: CTATACACAGACTCGCTGTCAC<br>R: AAGACTCTCCAGGACCAGAT | 113 bp |
| *Bid* | F: TCACAGACCTGCTGGTGTTC<br>R: TGTCTGGCAATGTTGTGGAT | 220 bp |
| *Mcl-1* | F: CCTTTACTGTTGGCGTGTTATG<br>R: GGGTGAGAGTTCTAATGCAGAT | 109 bp |
| *Bim* | F: CTTCCATACGACAGTCTCAG<br>R: TCTTCAGCCTCGCGGTAATC | 142 bp |
| *Htra2* | F: CGTGGAATACATTCAGACCG<br>R: AAACTCCCTAAGGCGATCAG | 151 bp |
| *Smac* | F: TTTCCTGTCTTGGCTAACTC<br>R: CTCCGATTTCTGAGCAATAGG | 123 bp |
| *survivin* | F: GCTGTACCTCAAGAACTACCG<br>R: GTTCCCAGCCTTCCAATTCC | 173 bp |
| *p53* | F: GGGAATAGGTTGATAGTTGTCAG<br>R: GGGTGAGATTTCATTGTAGGTG | 94 bp |

| | | |
|---|---|---|
| *Bak* | F: AGATGATATTAACCGGCGCTAC<br>R: GGCGATCTTGGTGAAGAGTT | 101 bp |
| *Xaf1* | F: CTGCGCTTCATAGTCCTTTGCC<br>R: CTTGGCAGGGTGCTGTTGG | 117 bp |
| *Bcl-w* | F: CAAGTGCAGGATTGGATGGTGG<br>R: CTGTCCTCACTGATGCCCAGTT | 157 bp |
| *Bad* | F: AGACGCTAGTGCTACAGATAGG<br>R: CGTCCCTGCTGATGAATGTT | 202 bp |
| *GAPDH* | F:CAATGTGTCCGTCGTGGATCT<br>R:GTCCTCAGTGTAGCCCAAGATG | 124 bp |

**Supplementary Table S3 Primers used for semi-quantitative PCR.**

| **Gene** | **Primer sequence (5′to 3′)** | **Product size** |
|---|---|---|
| *XIAP* | F: TCACTTGAGGTCCTGATTGC<br>R: GCTTGAACGTAATGACGGTG | 305 bp |
| *cIAP1* | F: GCACAGACAGTTCTATCCCA<br>R: GCTCTGACATAGCATCATCC | 351 bp |
| *survivin* | F: GCTGTACCTCAAGAACTACCG<br>R: GTTCCCAGCCTTCCAATTCC | 167 bp |
| *Smac* | F: TTTCCTGTCTTGGCTAACTC<br>R: CTCCGATTTCTGAGCAATAGG | 210 bp |
| *Htra2* | F: CGTGGAATACATTCAGACCG<br>R: AAACTCCCTAAGGCGATCAG | 298 bp |
| *Xaf1* | F: CTTCATAGTCCTTTGCCCAG<br>R: GATCTTCGATTTCCAGAGTCC | 317 bp |
| *Zbtb38* | F: CCATAGATCACAGACTCTCCAT<br>R: CTGTAGCTGATCACAGAGGCCGAG | 511 bp |
| *GAPDH* | F: CCATCACCATCTTCCAGGAG<br>R: CCTGCTTCACCACCTTCTTG | 577 bp |

**Supplementary Table S4 Primers used for ChIP assay.**

| **Location** | **Primer sequence (5′to 3′)** |
|---|---|
| **a** | F: GAACTTTGCCTTGAATATGTAATG<br>R: CAGAGCAGAATTACTGATC |
| **b** | F: CAAACAGATTGTTCCTTGACCAACAG<br>R: TGATGGAAGTGGACCATTCCT |
| **c** | F: CTATCATTATCCAGCTTATGCTGATTC<br>R: CACATATCTCTGAAGAGCTGAC |
| **d** | F: GGACGTAATCTTCATAATGTA<br>R: GTCACATAAAGGCGAACTTCC |
